# Comparison of reference gene expression stability in mouse skeletal muscle via five algorithms

**DOI:** 10.7717/peerj.14221

**Published:** 2022-10-17

**Authors:** Jianfeng Ma, Jingyun Chen, Mailin Gan, Lei Chen, Ye Zhao, Lili Niu, Yan Zhu, Shunhua Zhang, Xuewei Li, Zongyi Guo, Jinyong Wang, Li Zhu, Linyuan Shen

**Affiliations:** 1 Department of Animal Science, College of Animal Science and Technology, Sichuan Agricultural University, Chengdu, China; 2 Key Laboratory of Livestock and Poultry Multiomics, Ministry of Agriculture and Rural Affairs, College of Animal and Technology (Institute of Animal Genetics and Breeding), Sichuan Agricultural University, Chengdu, China; 3 Chongqing Academy of Animal Science, Chongqing, China; 4 College of Life Science, China West Normal University, Nanchong, China

**Keywords:** RT-qPCR, Reference gene selection, Skeletal muscle, Gene expression, Mice

## Abstract

Real-time quantitative PCR (RT-qPCR) is a widely applied technique for relative quantification of gene expression. In this context, the selection of a suitable reference gene (RG) is an essential step for obtaining reliable and biologically relevant RT-qPCR results. The present study aimed to determine the expression stability of commonly used RGs in mouse skeletal muscle tissue. The expression pattern of eight RGs (ACTB, GAPDH, HPRT, YWHAZ, B2M, PPIA, TUBA and 18S) were evaluated by RT-qPCR in different sample groups classified based on genetic background, muscle tissue type, and growth stage, as well as in a C2C12 myoblast cell line model. Five computational programs were included in the study (comparative ΔCq value, NormFinder, BestKeeper, geNorm, RefFinder) to evaluate the expression stability of RGs. Furthermore, the normalization effects of RGs in soleus (SOL) and gastrocnemius (GAS) muscle tissue were evaluated. Collectively, ACTB, HPRT and YWHAZ were shown to be the most stable RGs, while GADPH and 18S were the least stable. Therefore, the combined use of ACTB, HPRT and YWHAZ is recommended for the normalization of gene expression results in experiments with murine skeletal muscle. The results discussed herein provide a foundation for gene expression analysis by RT-qPCR in mammalian skeletal muscle.

## Introduction

In most mammals, nearly 40% of body weight is composed of skeletal muscle, which is a complex and heterogeneous tissue (Bentzinger, Wang & Rudnicki, 2012). Skeletal muscle plays an important role in locomotion and metabolic regulation of the body. Several physiological processes occur in skeletal muscle and involve changes in gene expression, such as myogenesis (Wang et al., 2019), atrophy (Yin et al., 2021) and regeneration (Tidball, 2017). Recent advances in molecular biology, including microarrays (Tarca, Romero & Draghici, 2006), quantitative polymerase chain reaction (qPCR) (Gibson, Heid & Williams, 1996) and RNA sequencing (RNA-Seq), have been employed in the determination of changes in gene expression in skeletal muscle. Thus, gene expression analysis is central for exploring the functions of candidate genes to advance biological research (Bustin, 2002).

Real-time quantitative PCR (RT-qPCR) is a commonly used method in gene expression analysis. Since its initial development in the early 1980s, PCR has been adapted for various applications in molecular cloning (Mullis, 1990). Heid et al. (1996) developed a RT-qPCR method that enabled measuring PCR product accumulation using a dual-labeled fluorogenic probe. Gene expression analysis usually involves RNA isolation, reverse transcription or cDNA synthesis, and RT-qPCR amplifications using fluorescent dyes (such as SYBR green). During cDNA synthesis, the fluorescent dye binds to double-stranded DNA and emits fluorescence. Thus, at the end of each thermal cycle during RT-qPCR, the intensity of fluorescence is measured. The cycle threshold (Ct) value is the number of cycles required for the fluorescence signal to surpass an established threshold and is inversely proportional to the amount of the target RNA in the sample, thus being used for quantifying gene expression. The Ct value is also known as cycle of quantification (Cq) (D’haene, Vandesompele & Hellemans, 2010), which correlates linearly with the logarithmic value of the initial number of copies of cDNA in the sample.

RT-qPCR is considered the gold standard for gene expression analysis owing to its specificity, sensitivity, accuracy and reproducibility (Bustin et al., 2005). It enables relative and absolute quantification of expression levels, but relative quantification is the most frequently used method (Bustin, 2002). Relative quantification of gene expression requires the adoption of reference genes whose expression levels are often stable across a variety of physiological conditions, being thus used to normalize RT-qPCR results of expression levels of target genes. In 2009, MIQE guidelines for the standardization of the evaluation of RT-qPCR experiments were published(Bustin et al., 2009), which emphasized the importance of validating reference genes (RGs) for gene expression analysis.

RGs are central for a proper understanding of the biological significance of RT-qPCR results. The selection of inadequate RGs may lead to inaccurate or wrong conclusions. Commonly, RGs are well-known housekeeping genes that participate in fundamental functions in cells (Schmittgen & Zakrajsek, 2000). Certain protein-encoding genes, such as ACTB, GAPDH and 18S, are regarded as having a stable expression, since they are required for basic cellular functions (Thellin et al., 1999). Brett et al. used ACTB, HPRT and GAPDH as RGs to study skeletal muscle repair (Brett et al., 2020, p. 1). Luo et al. (2021) carried out normalization for the gene coding for transmembrane protein 182 using GAPDH as RG in skeletal muscles. When studying muscle regeneration, Ding et al. (2021) adopted the 18S gene as RG. Thus, a unique criterion for the selection of RGs for the study of skeletal muscles is still lacking, which highlights the need for further investigation on the selection of RGs for skeletal muscle experiments.

A variety of computational tools have been developed to assess the suitability of RGs. Andersen, Jensen & Øntoft (2004) developed NormFinder, which enables the calculation of RGs stability according to intra-group and inter-group variation. The geNorm algorithm excludes unstable RGs to enable individual calculation of the expression stability (M value) of RGs (Vandesompele et al., 2002), and inadequate RGs are then discarded until two optimal RGs are found. Moreover, Pfaffl et al. (2004) developed BestKeeper to identify the most stable RGs based on the standard deviation (SD) and coefficient of variance (CV) of Cq value of RGs. In addition, Silver et al. (2006) proposed a comparative Ct value algorithm that enabled the calculation of the SD of the difference between Cq values (ΔCq) of two genes across all samples, and RGs are then ranked based on the average of SD (Andersen, Jensen & Øntoft, 2004). However, RefFinder is the most recent online tool for the analysis of the selection of RGs (Xie et al., 2012), which comprehensively considered multiple algorithms to provide a ranking of RGs. These are free tools that are readily accessible online, thus providing a great convenience for the analysis of RGs.

Previous studies have reported the selection of RGs in muscle tissue (Nakao et al., 2015; Hildyard, Finch & Wells, 2019). In the present study, the expression stability of eight RGs in mouse skeletal muscle tissue was compared using five different algorithms, *i.e.*, comparative Cq value ( ΔCq), geNorm, NormFinder, BestKeeper and RefFinder. The selected RGs were ACTB, GAPDH, 18S and HPRT which are commonly used in RT-qPCR experiments in skeletal muscle; in addition, YWHAZ, PPIA, TUBA and B2M were included in the experiments based on previous studies (Masilamani, Loiselle & Sutherland, 2014; Nakao et al., 2015; Niu et al., 2016; Hildyard, Finch & Wells, 2019). Differences in the stability of RGs in mice of different genetic backgrounds, namely inbred C57BL/6 and outbred ICR was compared. Furthermore, differences in the stability of RGs in skeletal muscle in different body sections and growth stages were identified. In addition, murine C2C12 myoblast cell line was included as an *in vitro* model of skeletal muscle. Finally, the normalization effect of optimized combination of RGs were determined to RT-qPCR analysis of the following genes: troponin I1 (TNNI1), troponin I2 (TNNI2), troponin C1 (TNNC1), troponin C2 (TNNC2) in soleus (SOL) and gastrocnemius (GAS) muscle tissue. Taken together, the results discussed herein provided an optimized protocol for the selection of RGs to be applied in future RT-qPCR experiments in skeletal muscle samples.

## Materials & Methods

### Ethics statement

All procedures described herein involving animals were approved by the Animal Ethical and Welfare Committee of Sichuan Agricultural University, China (approval number: 20220207), according to the guidelines of the Regulations on the Management of Laboratory Animal License (Ministry of Science and Technology, China, 2004).

### Sample collection

C57BL/6 and ICR male mice were purchased from Chengdu Dashuo Experimental Animal Co., Ltd. (Chengdu, Sichuan, China). All animals were housed individually in plastic cages and maintained in a dedicated animal room at 22 °C ± 3 °C and 40% humidity under a natural light cycle. During the experiments, the animals were housed on shavings and provided with free sufficient food and water. All mice were treated humanely and killed by ether asphyxiation to collect muscle samples.

C2C12 cells line were purchased from NATIONAL INFRASTRUCTURE OF CELL-LINE RESOURCE (NICR) (http://www.cellresource.cn). C2C12 myoblasts in natural proliferation without any treatment were collected based on a previous study conducted by our group (Gan et al., 2022).

In order to investigate the effects of different factors on expression stability of RGs, RT-qPCR analysis were conducted on samples of four distinct groups:

(i) group A included soleus muscle tissue (SOL) samples from 8-week-year-old mice of two mouse lineages (C57BL/6 mouse, *n* = 8; ICR mouse, *n* = 6);

(ii) group B included samples from tibialis anterior (TA), longissimus dorsi (LD), gastrocnemius (GAS), SOL from 8-week-year-old ICR mice (*n* = 6);

(iii) group C included samples of mice in different growth stages (based on days of age), namely immature ICR mice in Lactation (*n* = 12) and Adulthood ICR mice (all samples in group B, *n* = 24);

(iv) group D was based on sample group, and differences between the expression stability of RGs in skeletal muscle tissue (SM) (above muscle tissue samples, *n* = 44) and C2C12 myoblasts line (*n* = 24) were explored.

### RNA extraction and reverse transcription

Total RNA from skeletal muscle and C2C12 myoblasts were extracted using the TRIzol kit (TaKaRa, Dalian, China) according to the manufacturer’s instructions. RNA concentration and quality were determined using NanoDrop 2000 (Thermo Scientific, Waltham, MA, USA) (Table S3). PrimeScript™ RT reagent kit with gDNA Eraser (TaKaRa) was used to remove genomic DNA from 1,000 ng total RNA samples subsequently used for reverse transcription according to the manufacturer’s instructions. Reverse transcription was conducted in the following conditions: 42 °C for 2 min; then maintained at 4 °C; followed by reverse transcription at 37 °C for 15 min, then heated to 85 °C for 5 s, and maintained at 4 °C. All samples were stored at −80 °C until further analysis.

### RT-qPCR

RT-qPCR was performed using the TB Green^®^ Premix Ex Taq™ II (Tli RNaseH Plus) kit (TaKaRa, Code No.RR820Q) in a CFX96 real time PCR detection system (Bio-Rad, Richmond, CA, USA) according to the manufacturer’s instructions. Amplification program was as follows: pre-denaturation at 95 °C for 5 min, followed by 40 cycles at 95 °C for 10 s, then at 60 °C for 30 s according to the manufacturer’s instructions. For each sample, RT-qPCR was performed in duplicates to enable calculation of the average Cq value for each gene. Cq value in each sample and sample information are listed in Table S1. Primer sequences were designed using the Primer-BLAST software (https://www.ncbi.nlm.nih.gov/tools/primer-blast) (Table S2). Melting curve analysis was carried out to verify primer specificity (Fig. S1). Primer pairs were validated by making standard curves using a cDNA dilution series to assess amplification efficiency. Primer amplification efficiencies are shown in Table S2. All selected primer pairs displayed between 90–110% amplification efficiency. Relative gene expression of target genes was calculated using the 2^−ΔΔCq^ method according to Livak method (Livak & Schmittgen, 2001).

### Stability analysis of RGs

Table 1 provides information on candidate RGs from the National Center for Biotechnology Information (NCBI) (https://www.ncbi.nlm.nih.gov/gene). The comparative Cq value (ΔCq) method refers to Nicholas et al. (Silver et al., 2006). The implementation of NormFinder (https://www.moma.dk/normfinder-software) and BestKeeper (version 1.0, https://www.gene-quantification.de/bestkeeper.html) computational tools was conducted on Microsoft Excel. geNorm analysis was implemented using the web-based version (https://seqyuan.shinyapps.io/seqyuan_prosper/). RefFinder analysis was performed using the website (http://blooge.cn/RefFinder/). All computational tools are freely available for download from the corresponding developers website.

**Table 1:** Reference genes information from NCBI.

**Gene symbol**	**Nomenclature**	**Function**	**NCBI Gene ID**
18S	18S ribosomal RNA	Cytosolic small ribosome subunit, translation	19791
TUBA	Alpha-tubulin	Microtubules of the eukaryotic cytoskeleton	22142
PPIA	Peptidylprolyl isomerase A	Peptidyl-prolyl cis-trans isomerase activit	268373
GAPDH	Glyceraldehyde-3-phosphate dehydrogenase	Oxidoreductase in glycolysis and gluconeogenesis	14433
B2M	Beta-2 microglobulin	Beta-chain of major histocompatibility complex class I	12010
HPRT	Hypoxanthine guanine phosphoribosyl transferase	Generation of purine nucleotides through the purine salvage pathway	15452
ACTB	Actin, beta	Cytoskeletal structural protein, involved in cell structure, integrity, and intercellular signalling	11461
YWHAZ	Tyrosine 3-monooxygenase/tryptophan 5-monooxygenase activation protein, zeta polypeptide	Signal transduction by binding to phosphoserine-containing proteins	22631

### Statistical analysis methods

Spearman correlation analysis and data visualization were performed using GraphPad Prism 8 software (GraphPad Inc., San Diego, CA). Comparison of intergroup means was performed using the non-parametric Mann–Whitney test in GraphPad Prism 8 software. Differences were reported as statistically significant when *p* values were <0.01.

## Results

### Expression patterns of selected RGs

**Figure 1:**
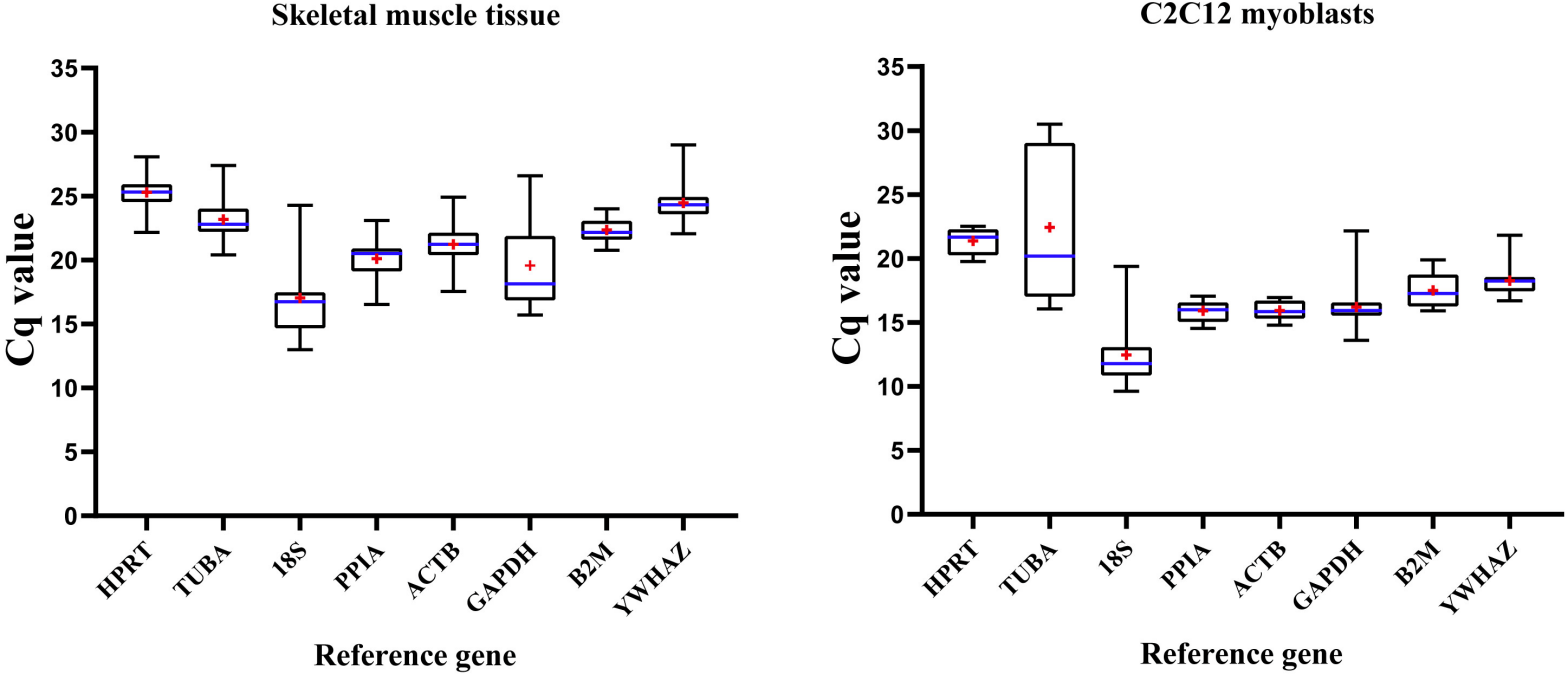
Distribution of RGs quantification cycle (Cq) values in murine skeletal muscle tissue (left) and C2C12 myoblasts (right). Boxes represent 25∼75% of data; whiskers indicate the minimum and maximum values of Cq values; blue lines indicate median values; + (red) indicates mean values.

Figure 1 illustrates expression patterns of raw Cq values of eight RGs in all mice skeletal muscle tissue and C2C12 myoblasts samples in present study. Cq values of RGs varied considerably; the lower the Cq value, the higher the number of the target transcript in the sample.

RGs were abundantly expressed in skeletal muscle tissue and C2C12 myoblasts (Cq values = 12.991∼28.675 and 9.623∼30.504, respectively), with similar expression patterns; 18S had the lowest Cq values in skeletal muscles (Cq value = 12.991∼24.282) and C2C12 myoblasts (Cq value = 9.623∼19.382). Cq values of HPRT, PPIA, B2M, ACTB and YWHAZ showed a lower extent of dispersion, while GADPH, TUBA, 18S showed a contrary pattern hence indicating that their expression pattern was unstable. Therefore, expression stability of RGs and the number of selected appropriate RGs were further explored. Then, we divided the samples into four groups to analyze the stability of RGs. Group A includes samples from different mouse strains. Group B includes samples from different skeletal muscular tissue types. Group C includes samples from different growth stages. The samples in group D were divided into skeletal muscle tissue or C2C12 myoblasts.

### Stability analysis of RGs by different algorithms

#### Comparative Cq value (ΔCq)

According to the results of ΔCq analysis, stability values of RGs in each sample group are shown in Table 2. The top two best RGs based on ΔCq results for each sample group was as follows: (i) group A: GADPH (0.38) and ACTB (0.40) in ICR, and ACTB (1.05) and B2M (1.09) in C57BL/6; (ii) group B: HPRT (0.45) and B2M (0.48) in TA, HPRT (0.57) and ACTB (0.58) in LD, HPRT (0.69) and B2M (0.69) in GAS, GAPDH (0.38) and ACTB (0.40) in SOL; (iii) group C: YWHAZ (0.91) and ACTB (1.03) in mice in lactation, HPRT (0.65) and YWHAZ (0.67) in adult mice; (iv) group D: YWHAZ (1.47) and TUBA (1.52) in skeletal muscle tissue, PPIA (1.89) and YWHAZ (1.90) in C2C12 myoblasts.

**Table 2:** Reference gene stability values for each group were obtained based on Comparative Cq ( ΔCq) analysis.

Group		HPRT	TUBA	18S	PPIA	ACTB	GAPDH	B2M	YWHAZ
Genetic background	ICR	0.46 (5)	0.47 (6)	1.03 (8)	0.69 (7)	**0.40 (2)**	**0.38 (1)**	**0.41 (3)**	**0.44 (4)**
	C57	**1.11 (4)**	**1.10 (3)**	2.59 (8)	1.25 (6)	**1.05 (1)**	1.80 (7)	**1.09 (2)**	1.12 (5)
Muscular tissue types	TA	**0.45 (1)**	**0.49 (3)**	1.34 (8)	0.52 (5)	0.54 (6)	0.57 (7)	**0.48 (2)**	**0.49 (3)**
	LD	**0.57 (1)**	**0.65 (4)**	1.24 (8)	0.72 (5)	**0.58 (2)**	0.92 (7)	0.72 (5)	**0.61 (3)**
	GAS	**0.69 (1)**	0.76 (6)	1.64 (8)	**0.73 (4)**	0.74 (5)	1.00 (7)	**0.69 (1)**	**0.72 (3)**
	SOL	0.46 (5)	0.47 (6)	1.03 (8)	0.69 (7)	**0.40 (2)**	**0.38 (1)**	**0.41 (3)**	**0.44 (4)**
Growth stage	Lactation	**1.04 (3)**	1.20 (6)	1.27 (7)	1.16 (5)	**1.03 (2)**	1.73 (8)	**1.05 (4)**	**0.91 (1)**
	Adulthood	**0.65 (1)**	**0.68 (3)**	1.36 (8)	0.83 (6)	**0.69 (4)**	0.94 (7)	0.72 (5)	**0.67 (2)**
Tissue or cell	SM	**1.60 (3)**	**1.52 (2)**	2.55 (7)	1.88 (6)	1.76 (5)	3.33 (8)	**1.65 (4)**	**1.47 (1)**
	C2C12	**2.08 (4)**	6.41 (8)	2.73 (7)	**1.89 (1)**	**1.94 (3)**	2.35 (5)	2.53 (6)	**1.90 (2)**
Geometric mean of ranks		**2.268**	4.315	7.686	4.703	**2.792**	4.710	**3.116**	**2.475**

**Notes.**

Values into the parenthesis refer to ranking of stability value in each group. Where 1 is given highest priority and 8 is the lowest priority. The last row is the geometric mean of the reference gene ranking in all groups. The top four RGs were marked in bold.

#### NormFinder

NormFinder is an application based on Microsoft Excel that considers intragroup and intergroup variations to calculate the stability of RGs. Table 3 shows expression stability values of RGs calculated based on NormFinder analysis. RGs with relative better ranking in each sample group were as follows: (i) group A: ACTB (0.068) and GAPDH (0.068) in ICR, TUBA (1.05) and YWHAZ (1.09) in C57BL/6; (ii) group B: YWHAZ (0.110) and HPRT (0.110) in TA, YWHAZ (0.221) and TUBA (0.275) in LD, YWHAZ (0.226) and TUBA (0.291) in GAS, ACTB (0.068) and GAPDH (0.068) in SOL; (iii) group C: YWHAZ (0.188) and B2M (0.456) in mice in lactation, YWHAZ (0.147) and TUBA (0.276) in adult mice; (iv) group D: YWHAZ (0.275) and TUBA (0.725) in skeletal muscle tissue, PPIA (0.166) and ACTB (0.166) in C2C12 myoblast cell line. The results obtained with NormFinder were similar compared with ΔCq results, with only a slight difference in ranking.

**Table 3:** Reference gene stability values for each group were obtained from NormFinder.

Group		HPRT	TUBA	18S	PPIA	ACTB	GAPDH	B2M	YWHAZ
Genetic background	ICR	0.280 (6)	0.244 (5)	1.011 (8)	0.654 (7)	**0.068 (1)**	**0.068 (1)**	**0.185 (4)**	**0.124 (3)**
	C57	0.902 (5)	**0.265 (1)**	2.562 (8)	1.130 (6)	**0.736 (3)**	1.465 (7)	**0.767 (4)**	**0.266 (2)**
Muscular tissue types	TA	**0.110 (1)**	0.315 (5)	1.329 (8)	0.413 (6)	0.465 (7)	**0.212 (3)**	**0.285 (4)**	**0.110 (1)**
	LD	**0.291 (3)**	**0.275 (2)**	1.186 (8)	0.607 (6)	**0.330 (4)**	0.740 (7)	0.582 (5)	**0.221 (1)**
	GAS	**0.523 (4)**	**0.291 (2)**	1.621 (8)	0.572 (5)	0.593 (6)	0.716 (7)	**0.517 (3)**	**0.226 (1)**
	SOL	0.280 (6)	0.244 (5)	1.011 (8)	0.654 (7)	**0.068 (1)**	**0.068 (1)**	**0.185 (4)**	**0.124 (3)**
Growth stage	Lactation	**0.737 (3)**	0.810 (5)	0.959 (6)	1.010 (7)	**0.767 (4)**	1.643 (8)	**0.456 (2)**	**0.188 (1)**
	Adulthood	**0.345 (3)**	**0.276 (2)**	1.304 (8)	0.677 (6)	**0.414 (4)**	0.703 (7)	0.498 (5)	**0.147 (1)**
Tissue or cell	SM	**1.091 (4)**	**0.275 (1)**	2.120 (7)	1.656 (6)	1.427 (5)	3.208 (8)	**0.617 (3)**	**0.275 (1)**
	C2C12	**1.082 (4)**	6.319 (8)	1.79 (6)	**0.166 (1)**	**0.166 (1)**	1.526 (5)	1.873 (7)	**0.427 (3)**
Geometric mean of ranks		**3.542**	**2.885**	7.453	5.158	**2.888**	4.328	3.896	**1.490**

**Notes.**

Values into the parenthesis refer to ranking of stability value in each group. Where 1 is given highest priority and 8 is the lowest priority. The last row is the geometric mean of the reference gene ranking in all groups. The top four RGs were marked in bold.

#### BestKeeper

BestKeeper evaluates the expression stability of RGs based on the *Pearson* coefficients of correlation (r), the coefficients of variation (CV) and standard deviation (SD) of Cq values. SD values of RG’s Cq values in each sample group are presented in Table 4. We then reanalyzed the *Pearson* coefficients of correlation between RGs with the BestKeeper index after excluding tow RGs with top SD for each group. ACTB and GAPDH were ranked as the most stable RGs in sample group of different genetic background. In TA, LD, GAS and SOL samples, top ranked RGs were YWHA, PPIA, ACTB and GAPDH, respectively. YWHAZ and HPRT were the most stable gene in samples of mice in the two growth stage (lactation: SD = 1.02, *r* = 0.984; adulthood: SD = 0.314, *r* = 0.888). In skeletal muscle tissue and C2C12 myoblasts, HPRT (SD = 0.942, *r* = 0.957) and YWHAZ (SD = 0.856, *r* = 0.919) were the optimal RGs, respectively. The performance of RGs based on Best-Keeper analysis was compared with ΔCq and NormFinder results, which revealed that low-ranking RGs were similar.

**Table 4:** Reference gene for each group were obtained from BestKeeper.

**Group**	**Genetic background**				**Muscular tissue types**								**Growth stage**				**Tissue or cell**			
	**ICR**		**C57**		**TA**		**LD**		**GAS**		**SOL**		**Lactation**		**Adulthood**		**SM**		**C2C12**	
	**SD**	**r**	**SD**	**r**	**SD**	**r**	**SD**	**r**	**SD**	**r**	**SD**	**r**	**SD**	**r**	**SD**	**r**	**SD**	**r**	**SD**	**r**
ACTB	0.447	0.979	0.683	0.981	0.133	0.951	0.19	0.87	0.556	0.987	0.447	0.979	0.787	0.942	0.332	0.78	1.25	0.955	0.676	0.721
HPRT	0.265	0.861	0.686	0.95	0.442	0.983	0.174	0.838	0.416	0.935	0.265	0.861	0.536	0.915	0.314	0.888	0.942	0.957	0.809	0.814
YWHAZ	0.541	–	1.133	0.919	0.597	0.989	0.252	0.001	0.321	0.299	0.541	–	1.02	0.984	0.469	0.625	1.086	0.894	0.856	0.919
B2M	0.259	0.981	0.635	0.967	0.259	0.914	0.382	0.743	0.452	0.951	0.259	0.981	1.058	0.781	0.443	0.858	0.768	0.73	1.245	0.541
PPIA	0.086	0.001	0.562	0.871	0.189	0.896	0.374	0.908	0.4	0.87	0.086	0.001	0.622	0.847	0.397	0.534	1.292	0.884	0.697	0.859
TUBA	0.385	0.886	0.999	0.94	0.266	0.907	0.274	0.227	0.469	0.698	0.385	0.886	1.507	0.935	0.386	0.702	1.156	0.854	5.381	–
GAPDH	0.423	0.993	2.228	–	0.719	–	0.683	–	0.638	–	0.423	0.993	2.213	–	0.701	–	2.892	–	1.309	0.902
18S	1.069	–	3.05	–	1.511	–	0.884	–	1.248	–	1.069	–	1.766	–	1.25	–	2.157	–	1.708	–

**Notes.**

SD Standard deviation of RGs r Pearson’s correlation coefficient between each RG and the BestKeeper index

#### geNorm

**Figure 2:**
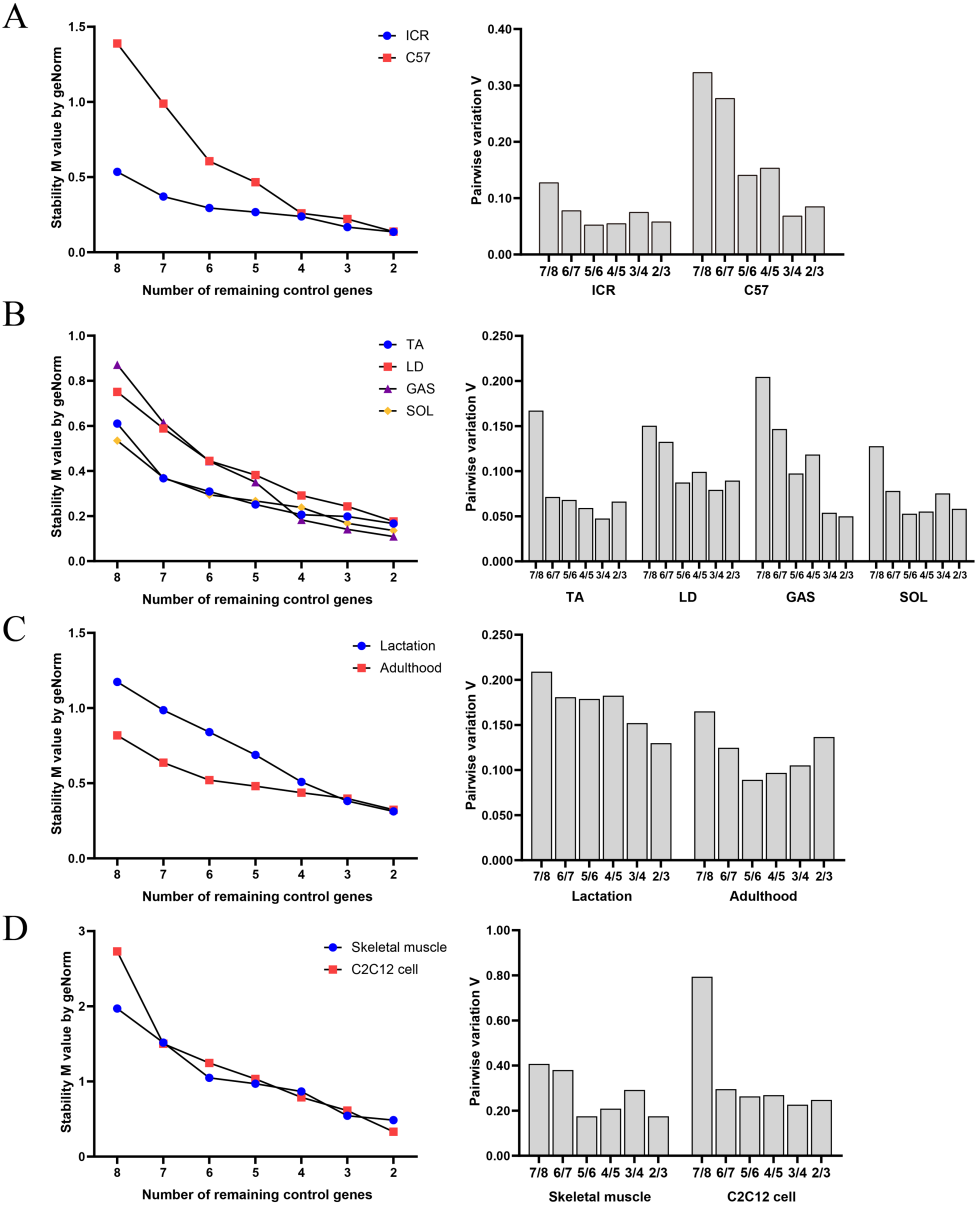
Average expression stability and optimal reference gene number obtained with geNorm algorithm. (A) Samples classified based on genetic background. (B) Samples classified based on muscle tissue type. (C) Samples classified based on growth stage. (D) Samples based on tissue or cell.

geNorm evaluated the stability of M values based on stepwise exclusion of the least stable RGs, and results are summarized in Fig. 2. RGs with high stability had lower M value. Table 5 shows the ranking of stability of RGs based on geNorm analysis. RGs with the highest suitability were as follows: (i) group A: GAPDH and YWHAZ in ICR, and HPRT and PPIA in C57BL/6; (ii) group B: TUBA and B2M in TA, HPRT and ACTB in LD, HPRT and PPIA in GAS, and GAPDH and YWHAZ in SOL; (iii) group C: PPIA and ACTB in mice in lactation, and HPRT and B2M in adult mice; (iv) group D: TUBA and YWHAZ in skeletal muscle tissue, and PPIA and ACTB in C2C12 myoblasts.

**Table 5:** Ranking of gene stability value by geNorm program.

**Group**	**Genetic background**		**Muscular tissue types**				**Growth stage**		**Tissue or cell**	
**Rank**	**ICR**	**C57**	**TA**	**LD**	**GAS**	**SOL**	**Lactation**	**Adulthood**	**SM**	**C2C12**
1	ACTB GAPDH	HPRT ACTB	TUBA B2M	HPRT ACTB	HPRT PPIA	ACTB GAPDH	PPIA ACTB	HPRT B2M	PPIA ACTB	PPIA ACTB
3	YWHAZ	B2M	ACTB	PPIA	B2M	YWHAZ	HPRT	ACTB	HPRT	HPRT
4	B2M	PPIA	PPIA	B2M	ACTB	B2M	YWHAZ	PPIA	YWHAZ	YWHAZ
5	TUBA	TUBA	HPRT	TUBA	TUBA	TUBA	B2M	TUBA	TUBA	B2M
6	HPRT	YWHAZ	YWHAZ	YWHAZ	YWHAZ	HPRT	TUBA	YWHAZ	B2M	GAPDH
7	PPIA	GAPDH	GAPDH	GAPDH	GAPDH	PPIA	18S	GAPDH	18S	18S
8	18S	18S	18S	18S	18S	18S	GAPDH	18S	GAPDH	TUBA

The ranking of RGs obtained with geNorm was similar compared to BestKeeper, NormFinder and ΔCq results. Pairwise variation of v values indicated that the use of two RGs is recommended for normalization of RT-qPCR results in each sample group.

#### RefFinder

**Figure 3:**
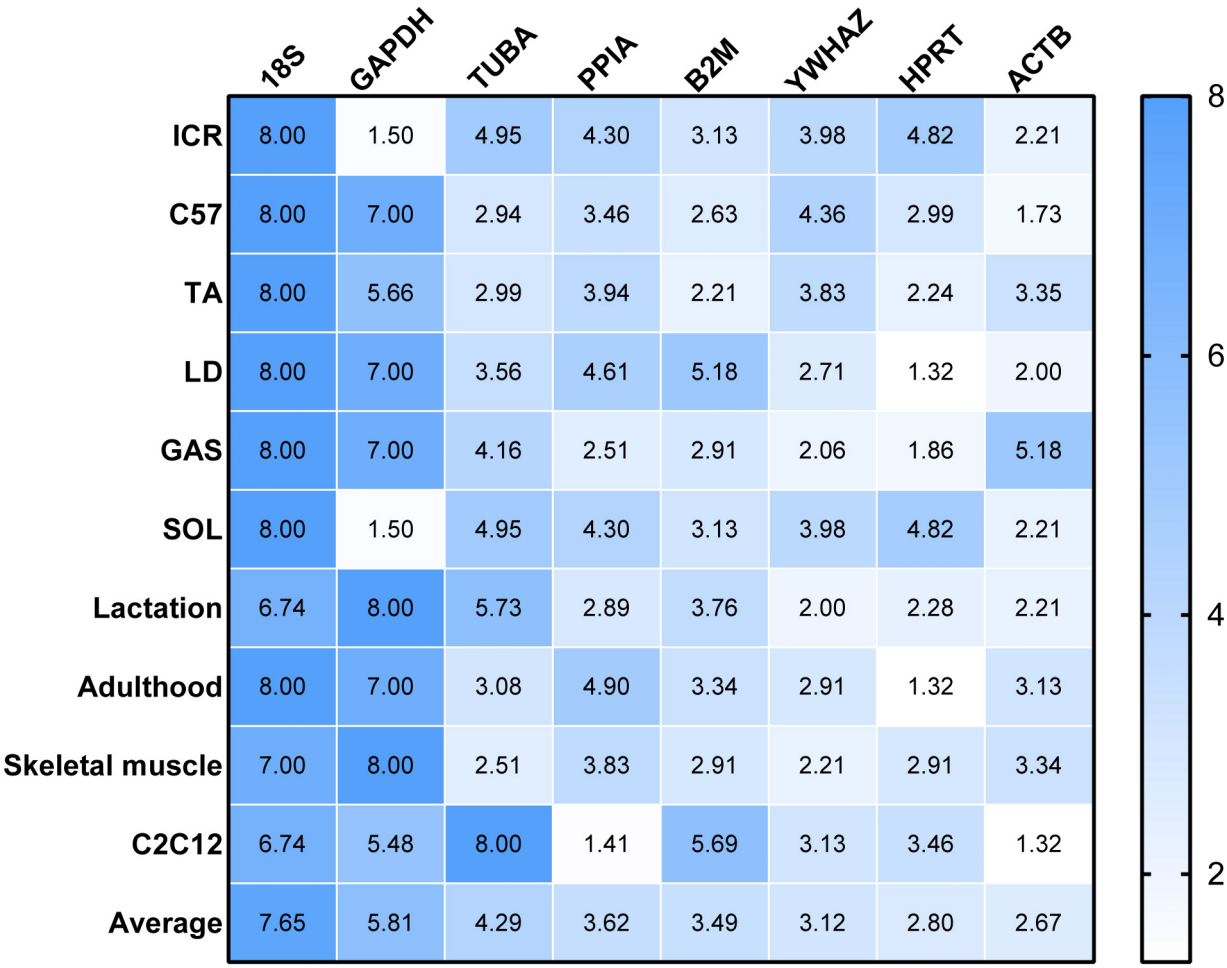
Comprehensive ranking values of reference genes in RT-qPCR experiments in murine models based on RefFinder analysis.

RefFinder was used to re-analyze the results of the other four algorithms to determine a ranking of RGs. The higher value of RGs form RefFinder denotes lower expression stability. In Fig. 3, a heatmap of the ranking of RGs based on RefFinder analysis is presented. Overall, RGs with the best ranking were ACTB (2.67), HPRT (2.80), YWHAZ (3.12) and B2M (3.49), whereas 18S (7.65), GAPDH (5.81), TUBA (4.29) and PPIA (3.62) performed poorly (Fig. 3).

### Spearman’s correlation analysis

To confirm the reliability of the five computational tools employed in the current study, Spearman’s correlation analysis was performed on the results of the ranking of RGs for each sample group. A strong correlation was found between the results obtained with comparative Cq value, NormFinder and RefFinder tools. Correlation coefficient be-tween ΔCq and NormFinder was 0.843; between RefFinder and ΔCq was 0.942; between NormFinder and RefFinder was 0.797. A moderate correlation was found in the following comparisons: ΔCq vs. BestKeeper (0.756), RefFinder vs. geNorm (0.747), ΔCq vs. geNorm (0.732). The lowest correlation coefficient was found between NormFinder and geNorm (0.501) (Table 6).

**Table 6:** Spearman correlation comparing all tools based on stability values ranking of reference gene.

	ΔCq	NormFinder	BestKeeper	geNorm	RefFinder
ΔCq	1	0.843192	0.756326	0.731880	0.942311
NormFinder	0.843192	1	0.559166	0.501354	0.797020
BestKeeper	0.756326	0.559166	1	0.719773	0.680952
geNorm	0.731880	0.501354	0.719773	1	0.747318
RefFinder	0.942311	0.797020	0.680952	0.747318	1

**Notes.**

All of *P* values were less than 0.05, which were considered to statistical significance correlation. This table omitted *P* value.

### RGs validation

**Figure 4:**
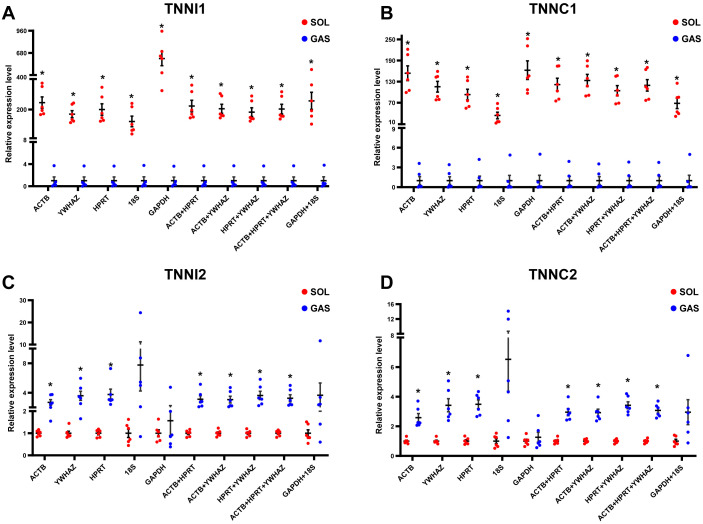
Effect of reference genes (RGs) normalization on gene expression of (A) TNNI1, (B) TNNC1, (C) TNNI2, and (D) TNNC2 in murine skeletal muscle tissue (soleus muscle, SOL; gastrocnemius, GAS). The expression of target genes was normalized based on the geometric means of different RGs combinations. (A) and (B): ^∗^*p* < 0.05, ^∗∗^*p* < 0.01 vs. the GAS group. (C) and (D): ^∗^*p* < 0.05, ^∗∗^*p* < 0.01 vs. the SOL group; *n* = 6. The results are expressed as mean ±standard deviation.

To evaluate the effect of normalization of RT-qPCR results based on the optimal RGs, a validation experiment was conducted in SOL and GAS. The expression of genes TNNI1, TNNC1, TNNI2, TNNC2 was normalized using RGs with high (ACTB, HPRT, YWHAZ) and poor performance (GAPHD and 18S) as demonstrated in the stability ranking ob-tained previously (Fig. 3). The combined use of ACTB, YWHAZ, HPRT yielded a more stable normalization of RT-qPCR results of the four target genes. Considering the normal-ization of TNNI1 and TNNC1, fold changes were higher using GAPDH as the RG, whereas an opposite trend was found when 18S was used as the RG (Fig. 4AB). No sig-nificant differences were found in the expression of TNNI2 and TNNC2 in SOL and GAS when using GAPDH as RG. A higher mRNA expression of TNNI2 and TNNC2 was observed in GAS when 18S was used alone as the RG (Fig. 4CD). Therefore, the combined use of ACTB, YWHAZ, HPRT as RGs was more suitable for the normalization of RT-qPCR results in murine skeletal muscle tissue than GADPH and 18S.

## Discussion

In the present study, five different algorithms were used to compared the expression stability of eight RGs in mouse skeletal muscle tissue. In addition, the effects of genetic background, skeletal muscle type and growth stages in the expression stability of selected RGs was compared. Furthermore, the C2C12 myoblast cell line, which has been widely used as an *in vitro* model to mimic skeletal muscle tissue, was included in the current study. Firstly, raw Cq values of RGs were evaluated; the higher the Cq values, the lower the abundance of nucleic acids. The distribution of Cq values of eight RGs indicated that 18S was the most abundant transcript in murine skeletal muscle and C2C12 myoblasts (Fig. 1). This is in agreement with another study employing mouse skeletal muscle tissue (Piazza et al., 2017). Cq values of all RGs evaluated herein were below 30, thus indicating that the expression levels of RGs were acceptable (Cedraz de Oliveira et al., 2017). Subsequently, RT-qPCR results were analyzed by ΔCq method and NormFinder. The results of ΔCq method and NormFinder were highly comparable, which showed that ACTB, YWHAZ, HPRT performed better (higher ranking) and 18S, PPIA, GAPDH performed poorly (lower ranking).

Based on the results obtained with BestKeeper, ACTB, HPRT, YWHAZ and B2M were higher-ranked, whereas GAPDH and 18S were lower-ranked. These results were not in complete agreement with those obtained with ΔCq and NormFinder, although poor-ranked RGs were comparable. This could be linked to the differences in the algorithm problem solving method. geNorm provided the ranking of RGs as well as the number of recommended RGs. The present results indicate that the two RGs met the recommended threshold, *i.e.*, V value <0.15 (Vandesompele et al., 2002). When V values are <0.15, it is not necessary to increase the number of RGs for normalization. However, V values >0.15 have also been reported in previous studies, which could be affected by the number of RGs and sample types (Kuijk et al., 2007). The accuracy of different tools influenced the selection of RGs. Thus, it can be hypothesized that there may have been a bias for the selection of RGs when using a single computational tool. Previous studies reported using multiple computational tools in combination of RGs selection (Fan et al., 2020; Jose et al., 2020). (De Spiegelaere et al., 2015) have reported that the result of RefFinder may be biased as it does not account for PCR amplification efficiences. Given this we calculate the geometric mean of the RGs ranking values obtained from above four methods to compare with RefFinder results (Fig. S2). This result is similar to the RefFinder result. The ranking of RGs based on the results of ΔCq, NormFinder, BestKeeper, geNorm, and comprehensively of RefFinder. Collectively, ACTB, HPRT and YWHAZ were considered the RGs with the best performance, whereas 18S, GAPDH and TUBA performed poorly.

Conversely, slight differences in ranking of RGs were observed between different sample groups. When considered the two different genetic backgrounds evaluated in the study, the most stable RGs were ACTB and GAPDH in ICR mouse, whereas ACTB and TUBA in C57BL/6 as indicated by ΔCq and NormFinder; in contrast, GAPDH and ACTB were found to be the most stable RGs both in ICR and C57BL/6 by BestKeeper. Using geNorm, ACTB was the most stable RG in the two genetic backgrounds. Kristen et al. (Thomas et al., 2014) reported that the stability ranking of RGs differed in three mouse strains (R129, C57BL/6J and C57BL/10). Thus, our results indicated that differences linked to mice genetic backgrounds were not significant when conducting RT-qPCR experiments on samples of the same tissue, and the stability of good RGs was similar among samples.

Additionally, the effect of muscle tissue type (TA, LD, GAS and SOL) on the stability of RGs was evaluated. In ΔCq analysis, the difference of stability between good RGs in TA and in GAS was low, HPRT = 0.45, B2M = 0.48, YWHAZ = 0.49, TUBA = 0.49, and HPRT = 0.69, B2M = 0.69, YWHAZ = 0.72, TUBA = 0.73, respectively. Thus, based on ΔCq and NormFinder results, HPRT and YWHAZ were considered, respectively, the optimal RGs in TA, LD and GAS, whereas ACTB and GAPDH were considered better RGs in SOL. SOL is a slow-twitch muscle fiber type, whereas TA, LD, GAS are fast-twitch muscle fiber types, which may have accounted for the observed discrepancies. Based on BestKeeper and geNorm results, high-ranked RGs were inconsistent in different skeletal muscles. Previous studies have focused on the selection of RGs in single skeletal muscle types (Thomas et al., 2014; Niu et al., 2016; Hildyard, Finch & Wells, 2019). The results discussed herein suggest that there is a difference on RGs stability in various murine skeletal muscle types.

Considering growth stage (mice in lactation and adult mice), HPRT, ACTB and YWHAZ were among the top four high-ranked among the five algorithms evaluated in the present study. Niu et al. (2016) reported candidate RGs in porcine skeletal muscle in 26 different developmental stages. Moreover, it has been suggested that the commonly used RGs GAPDH and ACTB may be not suitable for RT-qPCR experiments in skeletal muscle in different growth stages. Another study described that PPIA and HPRT were the most stable RGs in porcine longissimus dorsi muscle tissue in different developmental stages (Feng et al., 2010). Furthermore, C2C12 myoblast cell line was used herein as an *in vitro* cell model to study the stability of RGs in skeletal muscle; collectively, PPIAA, ACTB, HPRT, YWHAZ were more stable in C2C12 myoblasts, whereas YWHAZ, TUBA, HPRT, B2M were more stable in skeletal muscle tissue.

Considering all the above findings, ACTB, HPRT, YWHAZ, GAPDH and 18S were selected as RGs to normalize RT-qPCR results of target genes in SOL and GAS, which included TNNI1, TNNC1, TNNI2 and TNNC2. Troponin is the sarcomeric Ca2+ regulator for striated muscle contraction. TNNI1 and TNNC1 are exclusively expressed in slow skeletal muscle fiber types, while TNNI2 and TNNC2 are in fast skeletal muscle fiber types (Gomes, Potter & Szczesna-Cordary, 2002). The normalization of gene expression analysis based on the top ranked RGs (ACTB, HPRT and YWHAZ) yielded more replicable results. When normalizing TNNI1 and TNNC1, a significant difference was observed in SOL and GAS when using GADPH and 18S as RGs, although fold changes were discrepant. When normalizing TNNI2 and TNNC2, no significant differences were found in SOL and GAS using GADPH as RG. John et al. (Hildyard, Finch & Wells, 2019) found that normalization using GADPH yielded conflicting results in a mouse model of Duchenne muscular dystrophy compared with normalization conducted based on other RGs. This suggests that it may be not appropriate to conduct normalization of target genes in RT-qPCR experiments in skeletal muscle tissue using GADPH as the RG.

## Conclusions

In the present study, the expression stability of eight RGs in mouse skeletal muscle tissue was evaluated by five different computational tools. A strong correlation was found among the results obtained with ΔCq, NormFinder and RefFinder. Thus, a joint analysis of these tools is proposed to enable the proper selection of RGs for similar gene expression experiments. More specifically, based on the data discussed herein, the selection of ACTB as a RG in murine skeletal muscle tissue experiments may be the best choice for more reliable results of gene expression analysis. Moreover, if the experimental conditions permit, the combined use of ACTB, HPRT and YWHAZ could be considered the best option to normalize expression levels of target genes in murine skeletal muscle tissue experiments. The present study provides a useful guide for the selection of RGs for RT-qPCR experiments using murine skeletal muscle tissue.

##  Supplemental Information

10.7717/peerj.14221/supp-1 Supplemental Information 1 Amplification and dissolution curves

10.7717/peerj.14221/supp-2 Supplemental Information 2 Comprehensive ranking values of reference genes in RT-qPCR experiments in murine models based on geometric mean from Δ Cq, NormFinder, BestKeeper and geNorm

10.7717/peerj.14221/supp-3 Supplemental Information 3 Raw Cq values

10.7717/peerj.14221/supp-4 Supplemental Information 4 Primers sequence

10.7717/peerj.14221/supp-5 Supplemental Information 5 RNA quality

10.7717/peerj.14221/supp-6 Supplemental Information 6 Author Checklist - Full

## Additional Information and Declarations

### Data Availability

The following information was supplied regarding data availability: The raw measurements are available in the Supplementary Files.
