# Supplementary figures and images for: Comparison of reference gene expression stability in mouse skeletal muscle via five algorithms
Source: PeerJ. 2022 Oct 17;10:e14221. doi: 10.7717/peerj.14221 (PMC9583855; doi:10.7717/peerj.14221)

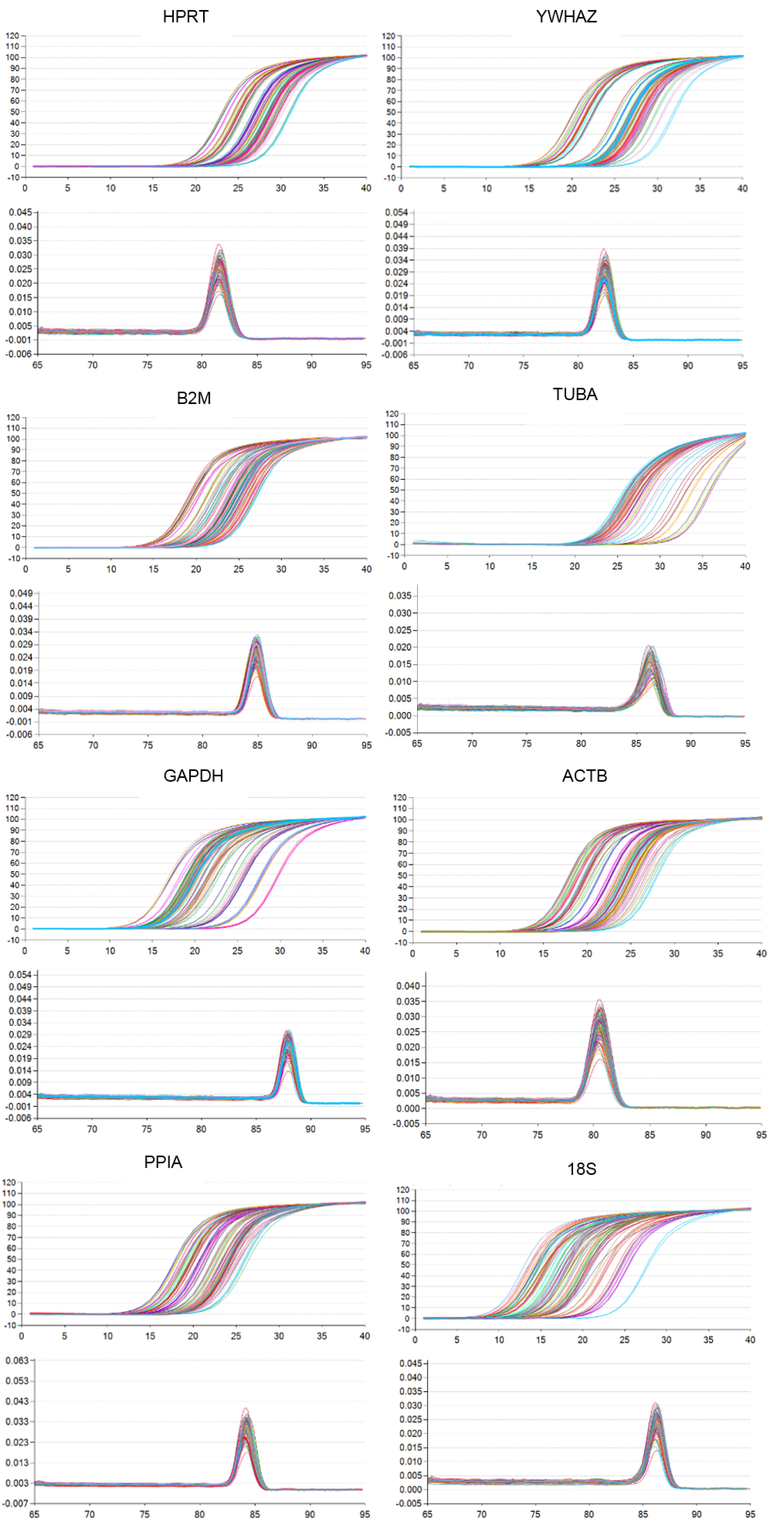

Supplement: Supplemental Information 1 [file peerj-10-14221-s001.jpg]

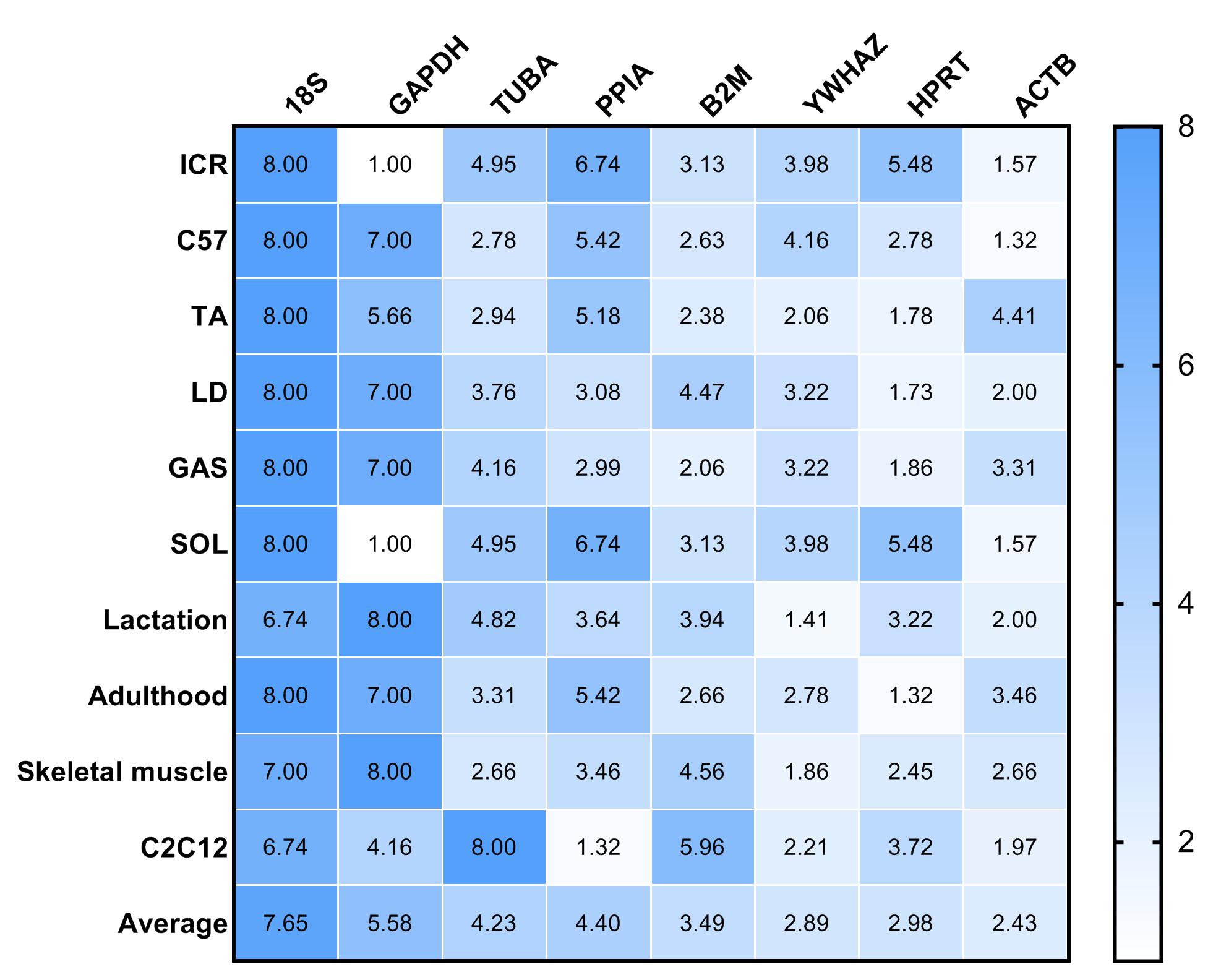

Supplement: Supplemental Information 2 [file peerj-10-14221-s002.jpg]
